# Risk of cardio-respiratory abnormalities in preterm infants placed in car seats: a cross-sectional study

**DOI:** 10.1186/1471-2431-5-28

**Published:** 2005-07-21

**Authors:** Vallier C Ojadi, Anna Petrova, Rajeev Mehta, Thomas Hegyi

**Affiliations:** 1 Department of Pediatrics, Division of Neonatal Medicine, Robert Wood Johnson Medical School / University of Medicine and Dentistry of New Jersey, New Brunswick, New Jersey, U.S.A

## Abstract

**Background:**

Little is known about the factors that predispose to the occurrence and severity of cardio-respiratory symptoms during the placement of a prematurely born infant in a car seat. The impact of gestational age, weight at discharge and infant's pre-existing cardio-respiratory status (in the supine position) on cardio-respiratory function during pre-discharge testing in a car seat (semi-upright position) has not been investigated.

**Methods:**

The cardio-respiratory function of 42 preterm neonates with gestational age 24 to 35 weeks and discharge weight 1790 to 2570 grams were monitored for 45 minutes before, during, and after placement in a car seat. The occurrence of periodic breathing, apnea, bradycardia, or decreased oxygen saturation (SaO2) was analyzed.

**Results:**

Prior to the car seat testing, 15 (35.7%) infants displayed one or more abnormalities of cardio-respiratory function. During the car seat testing, 25 (59.6%) infants had periodic breathing, 33 (78.2%) had oxygen saturation <90%, 14 (33.3%) had bradycardia less than 80 beats per minute, and 35 (83.3%) had a combination of these symptoms. Infants, both with and without pre-existing cardio-respiratory abnormalities, had an almost equal probability (80% vs. 83.3%) for the development of cardio-respiratory symptoms during placement in the car seat. Weight at discharge ([less than or equal to] 2,000 grams) but not the gestational age (<28 weeks or [greater than or equal to] 28<37 weeks), was associated with either increased episodes of oxygen desaturation or the combination of cardio-respiratory symptoms that were seen during the placement of these infants in the car seat. Repositioning from the car seat to the supine position showed normalization of cardio-respiratory function in the majority (83%) of the tested infants. None of the tested clinical factors were associated with the severity of the cardio-respiratory symptoms.

**Conclusion:**

Pre-discharge testing of the cardio-respiratory function of preterm infants during placement in a car seat is important for the prevention of cardio-respiratory symptoms during their transportation. However, the high risk for developing cardio-respiratory symptoms will require the consideration of an alternative mode of safe home transportation for preterm infants; especially those with a discharge weight less than 2,000 grams.

## Background

The American Academy of Pediatrics (AAP) recommends pre-discharge testing of cardio-respiratory function during placement in a car seat for all infants less than 37 weeks in order to identify the safe mode of home transportation [1]. Several studies have shown that cardio-respiratory function is compromised in 18.4% to 30.0% of preterm infants tested in a car seat [2-6] possibly due to excessive head flexion leading to restriction of the upper airway [7]. Preterm infants are particularly prone to head flexion when they are placed in an upright position and therefore more vulnerable to hypoxia and apnea when their neck is flexed [8].

Despite this improved knowledge, many questions regarding the safe transport of preterm infants remain unresolved. There is wide inter-hospital variation in the duration of car seat testing and the recommendations for infants who fail the test [9]. The risk factors that contribute to the development and severity of cardio-respiratory symptoms in preterm neonates during the car seat testing are still poorly defined. Only pre-existing apnea of prematurity is clearly recognized as a risk factor [5] and few others have been systematically studied.

Identification of neonates at high risk for intolerance to car seat testing is important in order to develop appropriate recommendations for a safe mode of home transportation. We hypothesized that the infant's maturity and cardio-respiratory status in the supine position would influence the risk as well as the severity of cardio-respiratory symptoms during pre-discharge car seat testing. The measurements recommended by the AAP [1] for the assessment of cardio-respiratory function of an infant placed in a car seat were included in the protocol. Additionally, the infant was monitored for periodic breathing because of its possible association with oxygen desaturation [10,11] as well as cyclical desaturation and reoxygenation of cerebral blood [12] in the supine position.

## Methods

A convenience sample of 42 preterm infants with gestational age ranging from 24 to 35 weeks (30.4 ± 3.3 weeks), birth weight of 550 to 2570 grams (1486 ± 523 grams), and discharge weight of 1790 to 2570 grams (2039 ± 215 grams) was studied 24–48 hours prior to discharge from the Neonatal Intensive Care Unit (NICU) at Saint Peter's University Hospital. The inclusion of 42 preterm infants would allow the detection of a thirty percent relative difference in the proportion of cardio-respiratory symptoms during placement in the car seat as compared to the supine position with a statistical power of 0.95 and Alpha <0.05 (two-sided).

Of the 42 sampled infants, 57.1% (n = 24) were white, 19.1% (n = 8) were black, 14.3 (n = 6) were Hispanic, and 9.5% (n = 4) were of other racial or ethnic groups; 4 (9.5%) were 24 weeks, 12 (28.6%) were 25 to 28 weeks, and 26 (61.9%) were 29–34 weeks gestation. The demographic characteristics of the study groups corresponded with the neonatal population discharged from the NICU at Saint Peter's University Hospital [13]. The study participants did not have any congenital anomalies, chronic cardiac, neurological or respiratory dysfunction, and at the time of testing none of them required oxygen supplementation or caffeine treatment for apnea of prematurity or any medications for gartroesophageal reflux. During the study period three of the discharged infants were not asked to participate in this study because one of the above mentioned conditions had been diagnosed during the NICU stay.

The study was approved by the Institutional Review Board (IRB) at the Robert Wood Johnson Medical School and the Saint Peter's University Hospital Committee for the Protection of Human Subjects in Research. Participation in the study required an informed consent to be signed by the parents. None of the mothers refused their baby's participation in the study.

### Study procedures

The study procedure included automatic monitoring of heart rate (HR), respiratory rate (RR), and oxygen saturation (O_2 _Sat) for 45 minutes in each of the three positions: regular bassinet (supine), followed by the car seat (semi-upright), and after return to the bassinet (supine). Infants were studied between feedings and left undisturbed during the testing. As in a previous study [3], the Cosco-Peterson First Ride car seat for infants (Cosco, Columbus, IN, USA) was used to construct the semi-upright position. ECG electrodes connected to a Health Dyne Smart 1600 monitor (Healthdyne Technologies, Marietta, GA, USA) interfaced with an Oxford Pneumocardiogram recorder (Oxford Medilog Systems, Abingdon, UK)) were used for the monitoring of the HR and RR. Oxygen saturation (%) was measured with a Nellcor Pulse Oximeter (Nellcor, Hayward CA, USA).

The infant's tolerance for the car-seat position was assessed by monitoring for apnea, bradycardia and decreased oxygen saturation that was based on accepted definitions as meeting at least 1 of the following criteria: apnea ≥ 20 seconds [6], bradycardia ≤ 80 beats per minute [14], or oxygen saturation <90% [15]. In addition, the monitoring and analysis of periodic breathing (defined as repeated ventilatory cycles with a clear respiratory pause of at least 2 seconds between cycles) was included in the study [16,10]. Severe cardio-respiratory symptoms were defined as a fall in oxygen saturation to below 80% [17] and a fall in heart rate to less than 70 beats per minute [18] respectively.

### Data analysis

The cardio-respiratory status prior to placement in the car seat (without or with any of the recorded abnormalities), the gestational age at birth (<28 vs. 28 to <37 weeks), and weight at discharge (< = 2,000 vs. >2,000 grams) were used to analyze the data. Postmenstrual age (PMA) at discharge was nearly identical across the gestational age groups and therefore was not used for the analysis. Chi-square and one-way ANOVA were used to compare categorical and continuous data, respectively. A conditional regression analysis was performed to evaluate the impact of gestational age, weight at discharge and the presence of cardio-respiratory symptoms in the supine position, on the infant's risk for developing cardio-respiratory symptoms during placement in the car seat. Statistical analysis was performed using Statistica 6.0 (StatSoft, Tulsa, OK, USA) and StatsDirect 2.4.4 (StatsDirect, Cheshire, UK). All data in the text are presented as mean ± standard deviation for continuous variables, as percentiles to identify the proportion, and crude as well as adjusted Odd Ratios (OR_C _and OR_A_) with 95% Confidence Interval (95% CI). P value <0.05 was considered statistically significant.

## Results

During testing in the supine position for 45 minutes before placement in the car seat, 35.7% (15/42) of the infants displayed one or more abnormalities (Table 1). The symptoms were generally mild and solitary except in the few infants (3/15) who had 2–3 documented episodes. The infants with cardio-respiratory symptoms recorded before placement in the car seat had been born at a lower gestational age and birth weight (28.0 ± 2.5 weeks and 1715 ± 500 grams, respectively) as compared to infants without cardio-respiratory symptoms (31.8 ± 2.8 weeks and 1105 ± 293 grams, respectively), P < 0.0001, but the weight at discharge was similar (1974 ± 175 vs. 2078 ± 230, P>0.05).

**Table 1:** Comparison of number of preterm neonates with cardio-respiratory symptoms before, during, and after placement in a car seat (n, %)

	Placement in the car seat				
	
Cardio-Respiratory Symptom	Before (1) (n = 42)	During (2) (n = 42)	After (3) (n = 42)	P_1–2_	P_2–3_
Periodic Breathing	9 (21.4%)	25 (59.5%)	2 (4.8%)	0.01	0.001
Apnea	0	2 (4.8%)	0	NS	NS
Bradycardia (HR ≤80/min)	4 (%)	14 (33.3%)	0	0.001	NS
O_2 _Saturation<90%	9 (21.4%)	33 (78.6%)	7 (16.7%)	0.01	0.01
Combination*	10 (23.8%)	35 (83.3%)	3 (7.1%)	0.001	0.001

* Presence of any cardio-respiratory symptoms

**Figure 1:**
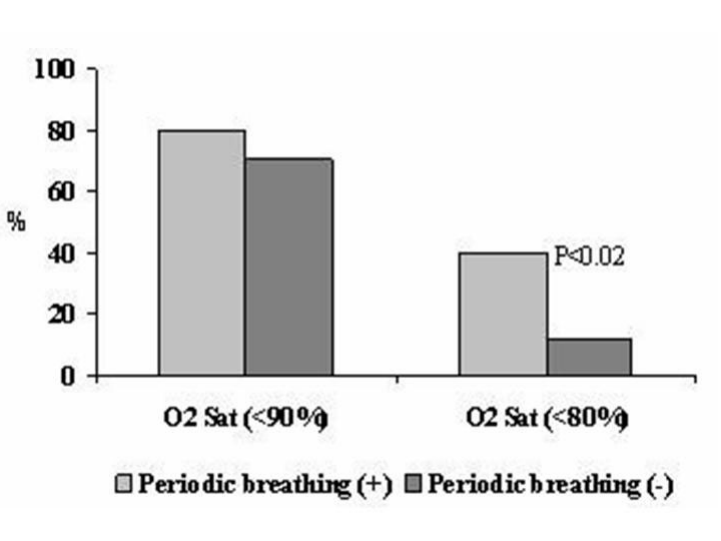
Periodic breathing associated Oxygen Desaturation (O2 Sat, %).

As shown in Table 1, cardio-respiratory symptoms were recorded with a greater frequency during placement in the car seat, and the majority of infants (83.3%, P < 0.001) showed a combination of cardio-respiratory symptoms. Placement of infants back in the supine position generally resulted in the resolution of the cardio-respiratory symptoms. During the car seat testing, oxygen desaturation <80% was observed in 13 (31.0%) of the tested infants and occurred more than 3 times in 11 (26.2%) infants. Bradycardia <70 beats per minutes was recorded in 4 (9.5%) of the infants, and 5 (11.9%) had multiple episodes. There was a significant association between periodic breathing and falls in oxygen saturation to less than 80% (P = 0.02, Figure 1).

As shown in Table 2, before placement in the car seat the cardio-respiratory symptoms were more frequent among infants with gestational age <28 weeks and pre-discharge weight < = 2,000 grams, but during the car seat testing cardio-respiratory events occurred more frequently in neonates with pre-discharge weight < = 2,000 grams. Conditional regression analysis showed that infants with a pre-discharge weight < = 2,000 grams were at greater risk for the occurrence of cardio-respiratory symptoms (OR_C _4.23, 95%CI 1.02, 25.0). The risk of cardio-respiratory symptoms increased when gestational age and cardio-respiratory symptoms in the supine position were taken into account but did not reach statistical significance (OR_A _11.6, 95%CI 0.94, 141.3). The infant's weight at discharge < = 2,000 grams did not affect the severity of the recorded cardio-respiratory symptoms during the car seat testing. Approximately one-third of the infants in both the discharge weight groups developed severe oxygen desaturation (<80%) and had multiple episodes (more than 3). Overall, only 2 infants with gestational age <28 weeks, pre-discharge weight < = 2,000 grams and no evidence of cardio-respiratory symptoms before placement in the car seat, developed severe apnea during their first 20 minutes of testing.

**Table 2:** The differences in cardio-respiratory symptoms before and during car seat testing in association with symptoms in supine position, gestational age and pre-discharge weight (n, %).

Cardio-respiratory symptom	Car seat testing	Symptoms while supine		Gestational age (weeks)		Pre-discharge weight (grams)	
		
		Symptoms (+) (n = 15)	Symptoms(-) (n = 27)	<28 (n = 16)	28–34 (n = 26)	< = 2,000 (n = 24)	>2,000 (n = 18)
Periodic Breathing	before	9 (60.0%)	0	7 (43.8%)	2 (7.7%) †	5 (20.8%)	4 (22.2%)
	during	9 (60.0%)	16 (59.3%)	9 (56.3%)	16 (61.5%)	16 (66.7%)	9 (50.0%)
Bradycardia (HR ≤80/min)	before	4 (26.7%)	0	2 (12.5%)	2 (7.7%)	3 (12.5%)	1 (5.6%)
	during	5 (33.3%)	9 (3.3%)	5 (31.3%)	9 (34.6%)	9 (37.5%)	5 (27.8%)
O_2 _Saturation <90%	before	9 (60.0%)	0	5 (31.3%)	4 (15.4%) †	8 (33.3%)	1 (5.6%) †
	during	13 (85.2%)	20 (74.1%)	12 (75.0%)	21 (80.8%)	20 (83.3%)	13 (72.2%)
Combination *	before	10 (66.7%)	0	6 (37.5%)	4 (15.4%) †	9 (37.5%)	1 (5.5%) †
	during	12 (80.0%)	23 (85.2%)	14 (87.5%)	21 (80.8%)	22 (91.7%)	13 (72.2%) ††

*Presence of any cardio-respiratory symptoms

† (P < 0.05) differences in frequency of cardio-respiratory symptoms in supine position in association with gestational age and pre-discharge weight.

†† (P < 0.05) differences in frequency of cardio-respiratory symptoms during car seat testing in association with symptoms in supine position, gestational age, and pre-discharge weight.

## Discussion

The following conclusions can be made from this study: (i) during the 45 minutes of pre-discharge car seat testing, the majority of preterm neonates are at risk for the development of mild and infrequent episodes of cardio-respiratory symptoms such as oxygen desaturation and periodic breathing; (ii) there is a higher risk for the occurrence of cardio-respiratory symptoms during car seat testing in infants with a pre-discharge weight < = 2,000 grams that seemed to be related to lower gestational age at birth and an elevated rate of symptoms when supine; (iii) the presence of periodic breathing significantly increases the risk for the development of severe hypoxic events (oxygen saturation <80%) in preterm neonates placed in the car seat; (iv) repositioning from the car seat to the supine position eliminates the cardio-respiratory symptoms in the vast majority of tested infants.

Our findings are in accord with other investigators who showed a high rate of cardio-respiratory abnormalities, especially the development of oxygen desaturation during car seat testing [2-4,19,20]. For the first time we report that severe oxygen desaturation in infants during car seat testing is associated with episodes of periodic breathing. Because of the transient immaturity of respiratory control [21], periodic breathing occurs frequently in preterm infants and has been shown to be associated with oxygen desaturation even in the supine position [10,11]. The association between periodic breathing and cyclical desaturation as well as reoxygenation of the cerebral blood in term infants has been previously reported [12]. Yamamoto et all showed that during apneic attacks in preterm infants, oxygen saturation <85% was associated with a reduction in the cerebral blood flow and cerebral oxygenation, measured indirectly using near infrared spectroscopy [22]. Although monitoring for periodic breathing was not included in the AAP recommendations for pre-discharge car seat testing of preterm infants [1], our results strongly suggest that it may be of considerable value in identifying infants at high risk for oxygen desaturation during home transport.

The following aspects of our study may limit the interpretation of the results: (i) infants with chronic cardiologic, neurological, or respiratory dysfunction were not included the study population, however, the vast majority of prematurely born infants are discharged from the NICU in a normal condition; therefore, the number of infants with such complications at discharge would be very small; (ii) the duration of the cardio-respiratory events was not included in the results, because a wide variation was observed in the range (between 10–20 seconds) for both bradycardia and oxygen desaturation; and (iii) the stage and duration of sleep was not tested. A study showed that the frequency of adverse cardio-respiratory events increases during sleep [23]. There is delayed maturation and poor organization of sleep states in preterm infants [24-26]. Therefore, if preterm babies are left asleep in a car seat they are likely to be at an even greater risk for the occurrence of cardio-respiratory symptoms and ensuing episodes of oxygen desaturation. This is of particular concern since there is evidence that a high percentage of episodes of apnea and bradycardia are not detected clinically [27].

Our results may have important implications for the standardization of pre-discharge car seat testing protocols for preterm born infants. The findings of periodic breathing accompanied by oxygen desaturation during the pre-discharge car seat testing of preterm born infants should lead to the consideration of an alternative mode of home transportation. There is always the option of supine transport in a car bed. Nevertheless, newer approaches for the safe transportation of preterm born infants need to be explored. One possible approach has been described by Tonkin and his colleagues [19] who modified an infant car seat such that it allowed the infant's head to rest in the neutral position on the trunk and therefore prevented narrowing of the upper airway, which was associated with reduced frequency of oxygen desaturation in preterm infants restrained in car seats.

## Conclusion

Pre-discharge testing of the cardio-respiratory function of preterm infants during placement in a car seat is important for the prevention of cardio-respiratory symptoms during their home transportation. The findings of periodic breathing accompanied by oxygen desaturation during the pre-discharge car seat testing of preterm born infants should lead to the consideration of an alternative mode of home transportation; especially those with a discharge weight less than 2,000 grams.

## Competing interests

The author(s) declare that they have no competing interests.

## Authors' contributions

VO participated in the study design, data collection, and carried out the study procedure. AP performed the statistical analysis and drafted the manuscript. RM participated in the coordination, interpretation of the data and drafting of the manuscript. TH conceived the study and participated in its design. All authors read and approved the final manuscript.

## Pre-publication history

The pre-publication history for this paper can be accessed here:



## References

[B1] (1996). Safe transportation of premature and low birth weight infants : American Academy of Pediatrics Committee on Injury and Poison Prevention and Committee on Fetus and Newborn. Pediatrics.

[B2] Bass JL, Mehta KA, Camara J (1993). Monitoring premature infants in car seats implementing the AAP policy in the community. Pediatrics.

[B3] Willett LD, Leuschen MP, Nelson LS, Nelson RM (1989). Ventilatory changes in convalescent infants positioned in car seats. J Pediatr.

[B4] Bull MJ, Weber K (1988). Automotive restraint systems for premature infants. J Pediatr.

[B5] Willet LD, Leuschen MP, Nelson RM (1986). Risk of hypoventilation in premature infants in car seats. J Pediatr.

[B6] Bass JL, Mehta KA (1995). Oxygen saturation of selected term infants in car seats. Pediatrics.

[B7] Tonkin SL, Bennet L, Vogel S, Gunn AJ (2000). Apparent life-threatening events associated with positional airways obstruction in infancy. Pediatr Res.

[B8] Stark AR, Thatch BT (1976). Mechanisms of airway obstruction leading to apnea in newborn infants. J Pediatr.

[B9] Williams LE, Martin JF (2003). Car seat challenges: where are we in implementation of these programs?. J Perinat Neonatal Nurs.

[B10] Miller MJ, Carlo WA, DiFiore JM, Martin RJ (1988). Airway obstruction during periodic breathing in premature infants. J Appl Physiol.

[B11] Poets CF, Southall DP (1991). Patterns of oxygenation during periodic breathing in preterm infants. Early Hum Dev.

[B12] Urlesberger B, Pichler G, Gradnitzer E, Reiterer F, Zobel G, Muller W (2000). Changes in cerebral blood volume and cerebral oxygenation during periodic breathing in term infants. Neuropediatrics.

[B13] Petrova A, Mehta R, Anwar M, Hiatt M, Hegyi T (2003). Impact of race and ethnicity on the outcome of preterm infants below 32 weeks gestation. J Perinatol.

[B14] Martin RM, Abu-Shaweesh, Baird MT (2004). Apnea of Prematurity. Pediatr Resp Rev.

[B15] Barrington KJ, Finer NN (1990). Periodic breathing and apnea in preterm infants. Pediatr Res.

[B16] Graff M, Soriano C, Hiatt M, Hegyi T (1991). Undetected Apnea and bradycardia in preterm infants. Pediatr Pulmonol.

[B17] Poets CF, Stebbens VA, Alexander JR, Arrowsmith WA, Salfield SA, Southall DP (1992). Arterial oxygen saturation in preterm infants at discharge from the hospital and six weeks later. J Pediatr.

[B18] Southall DP, Richards JM, Rhoden KJ, Alexander JR, Shinebourne EA, Arrowsmith WA, Cree JE, Fleming PJ, Goncalves A, Orme RL (1982). Prolonged apnea and cardiac arrhythmias in infants discharged from neonatal intensive care units: failure to predict an increased risk for sudden infant death syndrome. Pediatrics.

[B19] Tonkin SL, McIntosh CG, Hadden W, Dakin C, Rowley S, Gunn AJ (2003). Simple car seat insert to prevent upper airway narrowing in preterm infants: A pilot study. Pediatrics.

[B20] Hertz G, Aggarwal R, Rosenfeld WN, Greensher J (1994). Premature infants in car seats: effect of sleep state on breathing. J Sleep Res.

[B21] Glotzbach SF, Ariagno RL, Beckerman RC, Brouillette RT, Hunt CE (1992). Periodic breathing:. Respiratory Control Disorders in Infancy and Children.

[B22] Yamamoto A, Yokoyama N, Yonetani M, Uetani Y, Nakamura H, Nakao H (2003). Evaluation of change of cerebral circulation by SpO2 in preterm infants with apneic episodes using near infrared spectroscopy. Pediatr Int.

[B23] Horne RSC, Sly DS, Cranage TM (2000). Effects of prematurity on arousal from sleep in the newborn infant. Ped Res.

[B24] Nagase H, Yonetani H, Uetani Y, Nakamura H (2002). Effects of child seats on the cardio-respiratory function of newborns. Pediatr Intern.

[B25] Simakajornboon N, Beckerman RC, Mack C, Sharon D, Gozal D (2002). Effect of supplemental oxygen on sleep architecture and cardiorespiratory events in preterm infants. Pediatrics.

[B26] Scher MS, Steppe DA, Dahl RE, Asthana S, Guthrie RD (1992). Comparison of EEG sleep measures in healthy full-term and preterm infants at matched conceptional ages. Sleep.

[B27] Razi NM, Humphreys J, Pandit PB, Stahl GE (1999). Predischarge monitoring of preterm infants. Pediatr Pulmonol.

